# Autoantibodies against Cytochrome P450 Side-Chain Cleavage Enzyme in Dogs (*Canis lupus familiaris*) Affected with Hypoadrenocorticism (Addison’s Disease)

**DOI:** 10.1371/journal.pone.0143458

**Published:** 2015-11-30

**Authors:** Alisdair M. Boag, Michael R. Christie, Kerry A. McLaughlin, Harriet M. Syme, Peter Graham, Brian Catchpole

**Affiliations:** 1 The Royal (Dick) School of Veterinary Studies, University of Edinburgh, Easter Bush Campus, Midlothian, United Kingdom; 2 Division of Diabetes & Nutritional Sciences, King’s College London, Hodgkin Building, Guy’s Campus, London, United Kingdom; 3 School of Life Sciences, University of Lincoln, Lincoln, United Kingdom; 4 Department of Clinical Sciences and Services, Royal Veterinary College, Hawkshead Lane, North Mymms, Hatfield, Hertfordshire, United Kingdom; 5 Faculty of Medicine and Health Sciences, University of Nottingham, Sutton Bonington, Leicestershire, United Kingdom; 6 Department of Pathology and Pathogen Biology, Royal Veterinary College, Hawkshead Lane, North Mymms, Hatfield, Hertfordshire, United Kingdom; GI Lab, UNITED STATES

## Abstract

Canine hypoadrenocorticism likely arises from immune-mediated destruction of adrenocortical tissue, leading to glucocorticoid and mineralocorticoid deficiency. In humans with autoimmune Addison’s disease (AAD) or autoimmune polyendocrine syndrome (APS), circulating autoantibodies have been demonstrated against enzymes associated with adrenal steroid synthesis. The current study investigates autoantibodies against steroid synthesis enzymes in dogs with spontaneous hypoadrenocorticism. Coding regions of canine *CYP21A2* (21-hydroxylase; 21-OH), *CYP17A1* (17-hydroxylase; 17-OH), *CYP11A1* (P450 side-chain cleavage enzyme; P450scc) and *HSD3B2* (3β hydroxysteroid dehydrogenase; 3βHSD) were amplified, cloned and expressed as ^35^S-methionine radiolabelled recombinant protein. In a pilot study, serum samples from 20 dogs with hypoadrenocorticism and four unaffected control dogs were screened by radio-immunoprecipitation assay. There was no evidence of reactivity against 21-OH, 17-OH or 3βHSD, but five dogs with hypoadrenocorticism showed immunoreactivity to P450scc compared with controls. Serum samples were subsequently obtained from 213 dogs diagnosed with hypoadrenocorticism and 110 dogs from a hospital control population. Thirty control dogs were randomly selected to establish a threshold for antibody positivity (mean + 3 × standard deviation). Dogs with hypoadrenocorticism were more likely to be P450scc autoantibody positive than hospital controls (24% vs. 1.2%, respectively; *p* = 0.0016). Sex was significantly associated with the presence of P450scc autoantibodies in the case population, with 30% of females testing positive compared with 17% of males (*p* = 0.037). Significant associations with breed (*p* = 0.015) and DLA-type (DQA1*006:01 allele; *p* = 0.017) were also found. This cross-sectional study indicates that P450scc autoantibodies are present in a proportion of dogs affected with hypoadrenocorticism.

## Introduction

Canine hypoadrenocorticism is characterised by a deficiency in production of corticosteroid hormones (usually cortisol and aldosterone) by the adrenal gland. The condition has been identified to have a moderate to severe impact on dog health and welfare affecting a wide range of popular breeds [1], and there is interest in the dog as a potential model of human disease [2,3]. Hypoadrenocorticism can be a challenging disease for veterinarians to diagnose; animals often present with waxing and waning non-specific clinical signs, including lethargy, anorexia, polyuria/polydipsia, vomiting and diarrhoea [4–7] that can become acutely life-threatening as a result of electrolyte disturbances [6,8,9]. Diagnosis of hypoadrenocorticism relies upon use of the ACTH stimulation test, whereby a deficiency in cortisol secretory capacity is demonstrated [5,10].

Dogs have a relatively high incidence of spontaneous hypoadrenocorticism, compared with other species, with reports of up to 100-fold higher disease prevalence compared with humans [5,8,11–13]. Some breeds of dogs (e.g. Portuguese water dogs, standard poodles and West Highland white terriers) show increased susceptibility to the disease, suggesting that genetic factors play a role [6,8,13–16]. Recent evidence supports an autoimmune pathogenesis for canine hypoadrenocorticism, with susceptibility linked to immune response genes including MHC class II, *CTLA4* and *PTPN22* [14,15,17–21]. Histopathology of adrenal glands from dogs affected with hypoadrenocorticism indicates lymphocytic adrenalitis leading to adrenocortical atrophy [22–26], suggesting an autoimmune pathogenesis similar in nature to human autoimmune Addison’s disease (AAD) [2]. Furthermore, use of indirect immunofluorescence has demonstrated the presence of adrenal autoantibodies in dogs affected with hypoadrenocorticism [23,27].

The presence of circulating autoantibodies is regarded as an important indicator of autoimmune disease [28–30]. In dogs affected with hypothyroidism, autoantibodies have been identified against thyroglobulin, thyroid peroxidase, thyroxine and triiodothyronine [31–35], similar to those seen in human lymphocytic thyroiditis [36,37]. There are differences in frequencies in autoantibodies in human and canine disease, and also between breeds in dogs. For example, thyroid peroxidase autoantibodies are found less commonly in dogs than man, with prevalence estimates for thyroglobulin autoantibodies of between 20 to 50%, and up to 85% in some breeds [31,32,37,38]. In canine diabetes mellitus, autoantibodies against insulin [39], proinsulin [40], GAD65 and IA-2 [41] have been documented, similar to the autoantibody specificities seen in human type I diabetes [42].

The presence of serum autoantibodies in human patients suffering from AAD has long been recognised [43]. The primary autoantigen in AAD appears to be 21-hydroxylase (21-OH), with specific autoantibodies present in around 90% of patients at diagnosis [44,45]. In addition, autoantibodies against 17-hydroxylase (17-OH), the cytochrome P450 side-chain cleavage enzyme (P450scc) and 3-β-hydroxysteroid dehydrogenase (3βHSD) have also been described [11,46,47].

The aims of the present study were to investigate whether antibodies against adrenal autoantigens, specifically enzymes of the corticosteroid synthesis pathway, are present in dogs affected with hypoadrenocorticism, and to assess the relationship between autoantibody status and clinical features of the disease.

## Materials and Methods

### Study population

Residual serum samples from dogs affected with hypoadrenocorticism, were collected, following completion of diagnostic testing, performed either by the Royal Veterinary College (RVC) Diagnostic Service (Hatfield, UK) or NationWide Laboratories (Poulton-le-Fylde, UK). Two hundred and thirteen dogs were identified as having hypoadrenocorticism, based on either clinical and diagnostic laboratory records that demonstrated cortisol deficiency (cortisol concentrations < 27.6 nmol/L) in the ACTH stimulation test with no known prior use of glucocorticoids (*n* = 150), or that had been diagnosed with hypoadrenocorticism previously and were being sampled for monitoring of steroid-replacement therapy (*n* = 63). For all samples, all other available laboratory records were assessed to allow the most robust phenotyping possible and dogs were only included if there was evidence that the clinician in charge of the case was satisfied of a diagnosis of hypoadrenocorticism and the dog was treated accordingly. Although not commonly measured, dogs that demonstrated low endogenous ACTH (indicative of secondary hypoadrenocorticism) were excluded. Twenty samples with ACTH stimulation test results consistent with a diagnosis of hypoadrenocorticism were selected for the initial pilot experiment, based on blood sampling within three months of diagnosis, and also to cover a range of breeds, ages and sex. Serum samples were obtained from a relatively small number of dogs (*n* = 4) affected with trilostane-associated (iatrogenic) hypoadrenocorticism, identified on clinical records including results of ACTH stimulation testing.

Serum samples were also collected from dogs affected with hyperadrenocorticism (*n* = 58) during ACTH stimulation testing for monitoring of treatment with trilostane (Vetoryl^®^, Dechra Veterinary Products Ltd., Sansaw, UK). Specific information was not available relating to the initial diagnosis, relying instead upon veterinary practitioners’ ability to make appropriate diagnostic, treatment and monitoring decisions. Serum samples from a hospital control population (*n* = 110) were residual following completion of diagnostic testing, and were collected from dogs with no prior history of endocrinopathy or immune-mediated disease.

The Royal Veterinary College Institutional Ethics and Welfare Committee approved the use of residual clinical samples, taken for diagnostic purposes, for research with informed owner consent. NationWide Laboratories has approval for utilising residual clinical samples for development of diagnostic assays, provided that UK data protection legislation is observed.

### Cloning of canine adrenal autoantigen genes and production of recombinant protein

Canine adrenal tissue was obtained from a dog undergoing routine necropsy, following euthanasia for non-adrenal disease, with informed owner consent for use of tissue for research purposes. Tissue was preserved in RNAlater^®^ (Ambion, Huntingdon, UK) prior to RNA extraction (GenElute™ Mammalian Total RNA Kit; Sigma-Aldrich, Poole, UK) and cDNA synthesis (ImProm-II™ Reverse Transcriptase, Promega, Southampton, UK) performed according to the manufacturer’s recommendations.

Primers (see the table in S1 Appendix) were designed to amplify coding sequences of selected canine adrenal autoantigen genes; *CYP21A2* (21-OH; NM_001003335.1), *CYP17A1* (17-OH; XM_535000.4), *CYP11A1* (P450scc; XM_535539.4) and *HSD3B2* (3βHSD; NM_001010954.2) by PCR from canine adrenal cDNA using Easy-A High Fidelity PCR Cloning Enzyme (Agilent Technologies, Santa Clara, CA, USA). Amplicons were cloned into the pSC-A vector (Agilent Technologies, Santa Clara, CA, USA), then sub-cloned into the pTNT™ (Promega, Southampton, UK) or pVAX1 vector in One Shot Top10Fʹ *E*. *coli* (Life Technologies, Paisley, UK). Plasmid DNA was isolated from recombinant bacterial cultures using the PureYield™ Plasmid DNA Miniprep Kit (Promega, Southampton, UK).

The TNT^®^ Quick Coupled Transcription/Translation System (Promega, Southampton, UK) was used to produce recombinant radiolabelled protein according to the manufacturer’s instructions. Briefly, reactions consisting of 40 μL Master Mix (containing T7 RNA polymerase for pVAX1 constructs or Sp6 RNA polymerase for pTNT constructs), 4 μL ^35^S labelled methionine (Perkin Elmer, Cambridge, UK), approximately 1 μg plasmid DNA, made up to 50 μL with molecular biology grade water were incubated for 90 min at 30°C in a water bath. The amount of radioactivity incorporated into 1 μL of the translated protein was determined by scintillation counting, following precipitation with 10% trichloroacetic acid (Sigma-Aldrich, Poole, UK).

### Analysis of recombinant protein products by SDS PAGE and autoradiography

Aliquots of radiolabelled translate representing each potential autoantigen (20,000 counts per minute; CPM) were diluted in 20 μL of SDS-PAGE loading buffer for separation by SDS-PAGE on 4–12% gradient gels with MES running buffer using the Invitrogen Bis-Tris mini-gel system (Life Technologies, Paisley, UK). After separation, proteins were fixed in the gel with methanolic acetic acid (20% methanol, 10% acetic acid) for 30 min and gels washed with water. Gels were then soaked in Kodak ENLIGHTNING™ Rapid Autoradiography Enhancer (Perkin Elmer, Cambridge, UK) for 30 min and dried on a vacuum gel dryer. The gel was then placed in an X-ray film cassette directly in contact with Kodak BioMax^®^ MR film (Sigma-Aldrich), with squares of Tracker Tape to help align the film with the gel after developing and left to expose at -80°C for 24 h. Films were then developed and aligned with the PAGE gel to determine relative molecular weights of the bands on the film.

### Radioimmunoprecipitation assay

The radioimmunoprecipitation assay was performed as previously described [41] with some modifications. Briefly, radiolabelled recombinant protein (20000 CPM radioactivity) was diluted in IMP dilution buffer (10 mM HEPES, 150 mM NaCl, 20 mM methionine, 10 mM benzimadine, 0.5 mg BSA, 2.5 mL 0.5% Triton X-100; all Sigma–Aldrich, Poole, UK) to give 20 μL per reaction, then filtered using a 22 μm filter prior to incubation with 10 μL serum in triplicate wells at 4°C overnight in V bottomed 96-well plates. In parallel, a separate 96-well opaque filter plate was blocked overnight with 100 μL/well of 2 mg/mL BSA in phosphate-buffered saline (PBS) and washed twice with IMP buffer prior to use. Ten microlitres of a 50% slurry of protein A sepharose beads (Sigma–Aldrich, Poole, UK) was added to each serum/translate mix and incubated with agitation for 20–25 min. The immunoprecipitate was carefully resuspended, transferred to the filter plate and washed five times with IMP buffer and once with molecular biology grade water. Following drying, 100 μL MicroScint™ (Perkin Elmer, Cambridge, UK) was added and radiation measured using a Chameleon™ V plate reader (Hidex, Turku, Finland). For each plate, the positive and negative controls, identified during initial screening, were in a standard position and all other samples for that plate were introduced in a random, coded order, without their disease status being identified.

The relative autoantibody reactivity was calculated to allow inter-assay normalisation of data as follows: (CPM_sample_—CPM_negative_standard_) / (CPM_high_standard—_CPM_negative_standard_) × 100. Negative and high standards were defined in the first screening experiments and used for all subsequent plates. All triplicates with a coefficient of variation (CV) greater than 0.5 were manually checked, intra- and inter- assay CVs were calculated on all data. The threshold value for autoantibody positivity was set at the mean + 3 × SD of controls (*n* = 30).

### Statistical analysis

Data were organised in Microsoft Excel 2010 version 14 (Microsoft Corporation, Redmond, WA, USA) and statistical analyses performed using SPSS Statistics for Windows, version 20.0 (IBM Corp, Armonk, NY, USA). GraphPad Prism version 6.02 (GraphPad Software Inc., CA, USA) was used for presentation of results. Categorical data were analysed using contingency tables, with Chi squared or Fisher’s exact test used for comparisons. Continuous data was tested for normality by manual inspection of histograms, Q-Q plots and Shapiro-Wilk [48]. For normally distributed data, comparisons were made using two-sided unpaired Student’s *t*-tests, or ANOVA with post-hoc Bonferroni correction for multiple comparisons. Data not normally distributed were analysed using the Mann-Whitney *U* test or Kruskal-Wallis H test. Significance was accepted at *p* < 0.05.

## Results

### Cloning and expression of canine adrenal antigens

**Fig 1:**
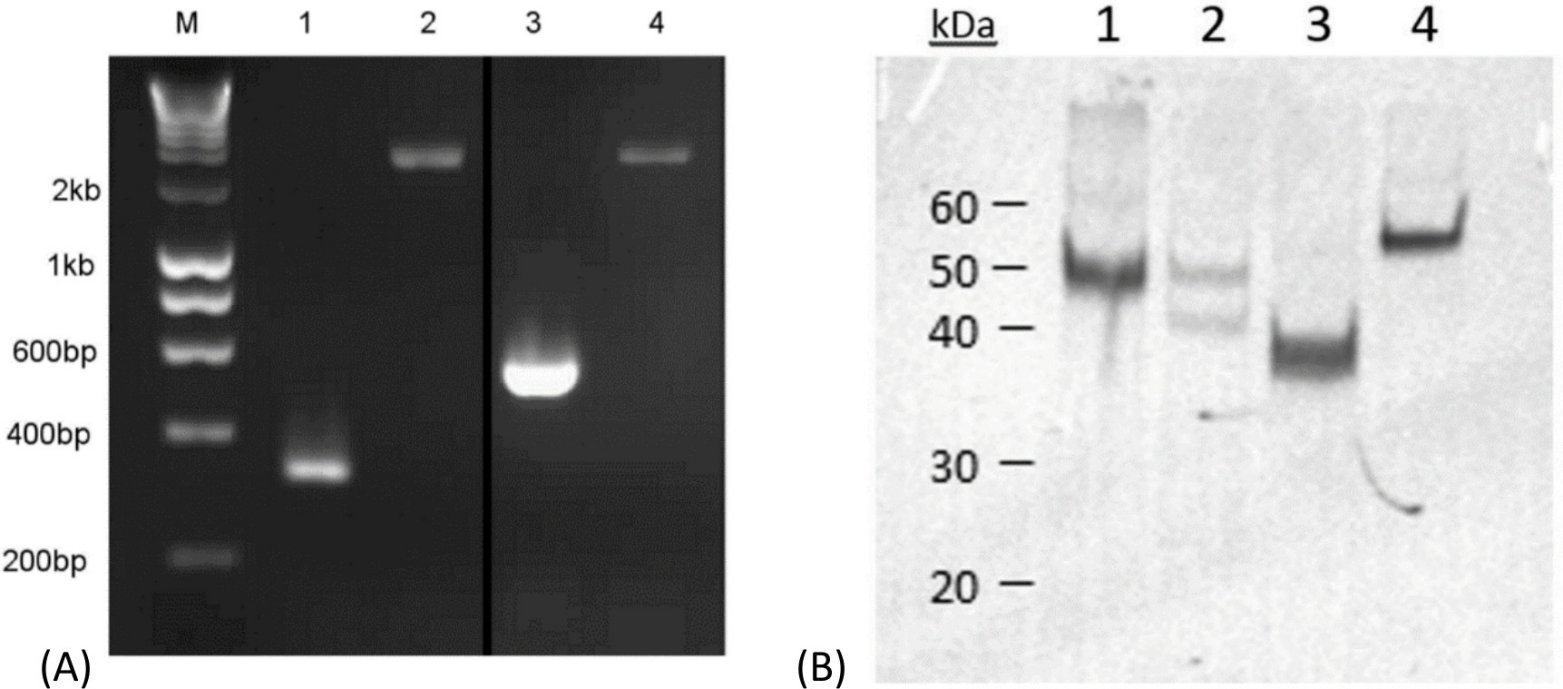
Amplification and expression of canine adrenal autoantigens. (A) Coding regions of selected canine adrenal steroid synthesis enzymes were amplified and cloned. Shown are PCR results of (1) screening primers and (2) cloning primers for canine *CYP17A1* and (3) screening primers and (4) cloning primers for canine *CYP11A1*. M, molecular weight ladder (Hyperladder I, Bioline, London, UK). Image has been cropped for ease of interpretation; the original image, with extra cDNA samples represented by extra lanes, is available as S1 Fig. (B) Autoradiograph of radiolabelled recombinant canine adrenal autoantigens. The coding regions of (1) canine *CYP21A2* (21-hydroxylase; 55 kDa), (2) *CYP17A1* (17-hydroxylase; 57 kDa), (3) *HSD3B2* (3β hydroxysteroid dehydrogenase; 42 kDa) and (4) *CYP11A1* (P450 side-chain cleavage enzyme; 60 kDa) were cloned into the pVAX1 vector and ^35^S-methionine radiolabelled recombinant protein expressed in an in vitro transcription and translation assay. Translates were subjected to SDS-PAGE and exposed to X-ray film for 24 h.

The selected genes (*CYP21A2*, *CYP17A1*, *CYP11A1* and *HSD3B2*) were successfully amplified from the canine adrenal cDNA (Fig 1A). Full length coding sequences were cloned into both pTNT and pVAX1 vectors and sequencing revealed each to be consistent with corresponding sequences in the CanFam3.1 dog genome assembly. (See: http://www.ncbi.nlm.nih.gov/genome/85) Use of recombinant plasmid DNA in the in vitro transcription and translation system demonstrated production of recombinant radiolabelled protein of the anticipated sizes for all constructs, but with an additional smaller band present for canine 17-OH, by autoradiography (Fig 1B).

### Screening of sera against adrenal antigens reveals P450scc autoantibodies

**Fig 2:**
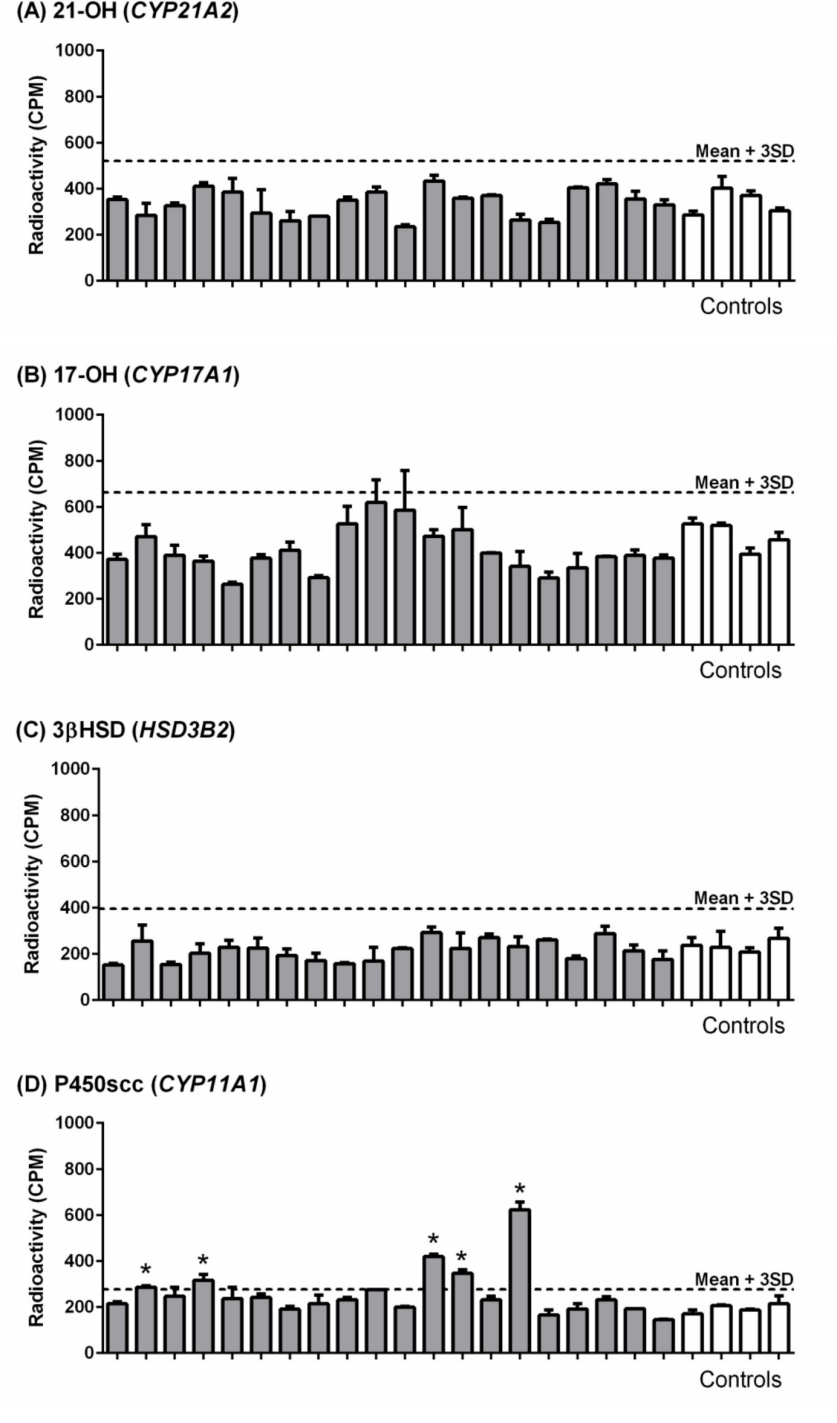
Radioimmunoprecipitation assays with canine sera against recombinant radiolabelled adrenal antigens. Radioactivity (counts per minute; CPM) for hypoadrenocorticism cases (*n* = 20; filled boxes) and control (*n* = 4; open boxes) serum samples after immunoprecipitation with recombinant canine (A) 21-OH; (B) 17-OH; (C) 3βHSD and (D) P450scc. Data are shown as mean + SEM. Hatched line represents positive threshold (mean + 3SD of control samples), with values above this indicated (*).

An experiment was performed to investigate autoantibody reactivity to the recombinant canine adrenal antigens with sera from dogs affected with hypoadrenocorticism (*n* = 20). These dogs were selected from all the samples collected based on blood sampling within three months of diagnosis, and covered a range of breeds, ages and sex. Control dogs (*n* = 4) had no known history of endocrinopathy, autoimmune disease or neoplasia. No difference in immunoreactivity was seen, comparing cases and controls for 21-OH, 17-OH and 3βHSD (Fig 2A–2C). In contrast, reactivity to P450scc was seen in a proportion of cases (Fig 2D), using the mean + 3SD CPM of the control values as the threshold for positivity. Five cases of hypoadrenocorticism were considered to be P450scc autoantibody positive, consisting of a German shepherd dog, beagle, lurcher, Great Dane and Polish lowland sheepdog, with an age of onset between 1 year and 4 years 7 months and comprising three females and two males.

### P450scc autoantibodies in dogs affected with adrenal disease

The P450scc radioimmunoprecipitation assay was optimised for construct design, expression conditions, type of precipitation, volume of serum and incubation times. It was found that using SP6 RNA polymerase with the canine *CYP11A1* sequence cloned into the pTNT vector produced the best yield of recombinant radiolabelled protein and that immunoreactivity was best determined with 10 min incubation of 10 μL serum with 20,000 CPM recombinant protein, subsequently precipitated with Protein-A sepharose.

To more fully characterise the P450scc autoantibodies in the dog population, sera were collected from four groups of dogs; a population of dogs affected with spontaneous hypoadrenocorticism (*n* = 213), dogs affected with hyperadrenocorticism (*n* = 58), dogs with iatrogenic hypoadrenocorticism following trilostane therapy (*n* = 4) and a population of hospital patients with no evidence of endocrine or immune-mediated disease (*n* = 110). Thirty of the hospital patients were randomly selected to act as the reference population to set the positive threshold value (mean ± 3SD); the other 80 dogs from this group acted as the control population for the autoantibody prevalence analysis.

**Fig 3:**
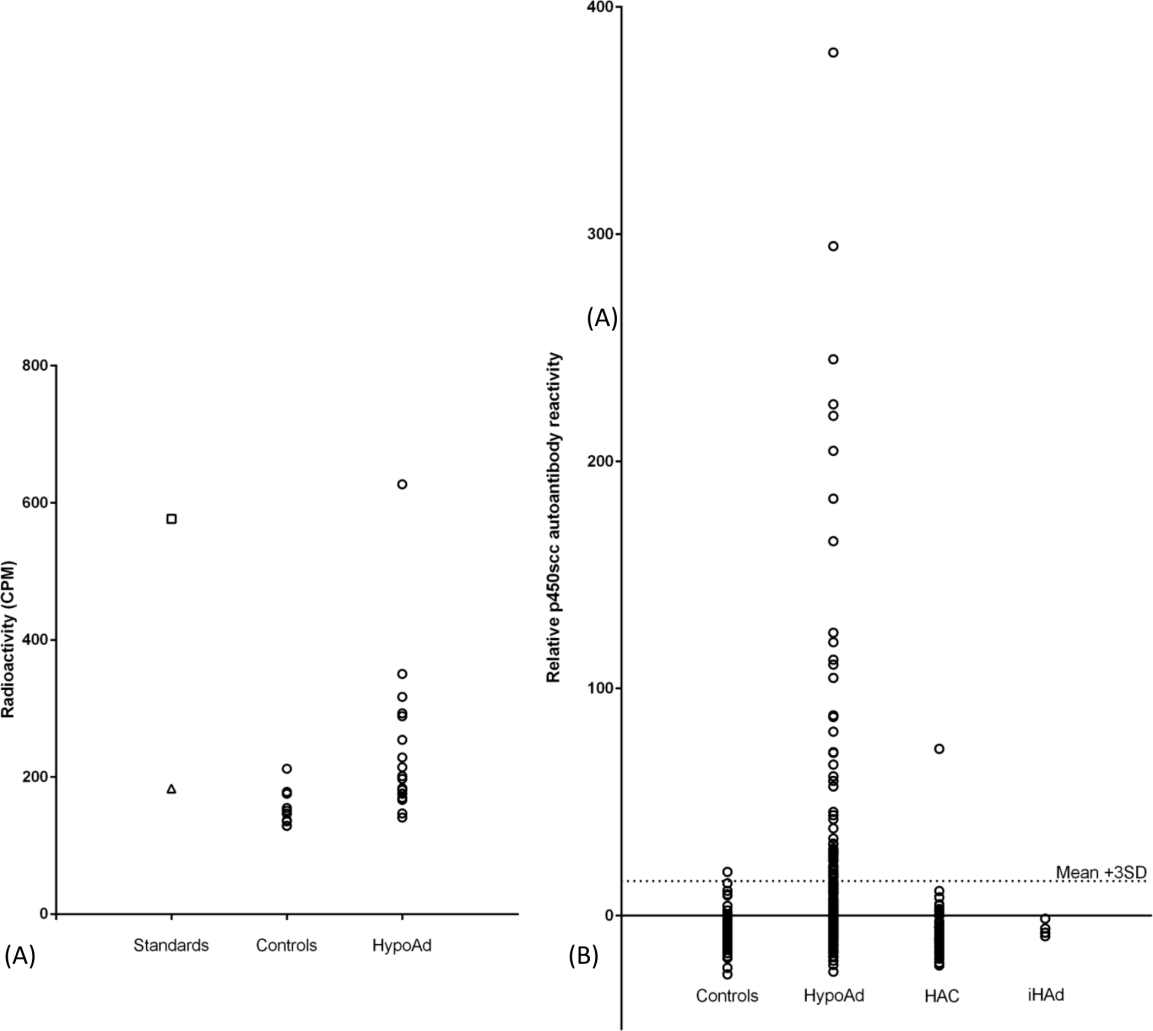
Serum antibodies to canine P450scc in radioimmunoprecipitation assay. (A) Scatter plot from a representative 96-well plate showing P450scc immunoreactivity (counts per minute; CPM, mean of triplicate wells) in serum samples from hospital control dogs (Control; *n* = 11) and dogs affected with hypoadrenocorticism (HypoAd; *n* = 19). Negative standard (triangle) and positive standard (square) serum samples were identified from initial autoantibody screening experiments and were used on every plate for normalisation purposes. (B) Relative P450scc autoantibody reactivity in control dogs (*n* = 80), dogs affected with spontaneous hypoadrenocorticism (HypoAd; *n* = 213), dogs affected with hyperadrenocorticism (HAC; *n* = 58) and dogs with iatrogenic hypoadrenocorticism (iHAd; *n* = 4). Circles represent normalised values for each individual serum sample in the P450scc radioimmunoprecipitation assay. Mean + 3SD line represents the threshold positive value established from a reference population of 30 canine control patients.

There was evidence for increased P450scc autoantibody reactivity in the dogs affected with hypoadrenocorticism (Fig 3A). Due to the large number of plates assessed, inter-assay variability was normalised using negative and positive control sera. There was significantly greater P450scc immunoreactivity in the dogs affected with hypoadrenocorticism compared with the hospital control population (*p* < 0.001; Fig 3B). There was no significant difference in autoantibody prevalence comparing the group of dogs selected on the basis of ACTH stimulation test results (n = 150) and those with a prior diagnosis of hypoadrenocorticism that were undergoing sampling for monitoring purposes (n = 63) and these two populations were combined for subsequent analysis. There was no significant difference comparing the hospital controls and the other two disease groups. Fifty one (24%) of the dogs affected with hypoadrenocorticism were considered to be positive for P450scc, based on the threshold set. One dog within the hospital control group, an 8 year old male Rottweiler affected with a sinonasal epithelial cell tumour, was considered to be weakly positive. There was one dog in the hyperadrenocorticism group that was considered positive, an 8 year old female neutered miniature poodle.

### P450scc autoantibody status and clinical parameters

Neither age at diagnosis nor time from diagnosis to sampling had a significant effect on autoantibody status. Sex was significantly associated with the presence of P450scc autoantibodies (*p* = 0.037), with 30% of affected female dogs testing positive, compared with 17% of males (odds ratio = 2.05, 95% confidence interval = 1.06–3.96). Breed classification was significantly associated with autoantibody prevalence (*p* = 0.015). Crossbreed dogs demonstrated P450scc antibody prevalence of 27%, similar to the population as a whole (24%), whereas West Highland white terriers had a lower autoantibody prevalence of 5% and collies had the highest prevalence of 50%.

### Relationship between dog leukocyte antigen (DLA) genes and P450scc autoantibody status

The differences in P450scc antibody status comparing breeds suggest that genetic factors might play a role in the response to this adrenal autoantigen. DLA haplotype information was available for 129 dogs [17]. Forty eight different haplotypes were represented, made up of 23 DLA-DRB1, eight DLA-DQA1 and 23 DLA-DQB1 alleles. No associations were found with P450scc autoantibody status across all haplotypes (*p* = 0.467), DLA-DRB1 (p = 0.142) or DLA-DQB1 (*p* = 0.222). However a significant association was found with DLA-DQA1*006:01 (*p* = 0.017; Table 1). A post-hoc analysis revealed that the haplotype DLA-DRB1*015—DQA*006—DQB1*023 containing this allele was associated with increased risk (OR = 2.36; 95% confidence interval = 1.21–4.60). No effect of DLA homozygosity was observed (*p* = 0.807).

**Table 1:** DLA-DQA1 association with P450scc autoantibody status.

DQA1 (n)	Number positive (%)	P value	Odds Ratio (95% CI)
001:01 (*n* = 77)	13 (16.9)	Baseline	Baseline
002:01 (*n* = 9)	2 (22.2)	0.653	1.407 (0.262–7.553)
003:01 (*n* = 14)	0 (0)	0.206	NaN
004:01 (*n* = 11)	0 (0)	0.162	NaN
005:01:1 (*n* = 16)	5 (31.3)	0.294	2.238 (0.665–7.532)
**006:01 (*n* = 78)**	**27 (34.6)**	**0.017**	**2.606 (1.223–5.557)**
009:01 (*n* = 22)	4 (18.2)	1	1.094 (0.318–3.768)
021:01 (*n* = 1)	0 (0)	1	NaN

Number (n) shown beneath each allele. Percentage positive in brackets next to the number positive. Significant association is shown in bold text.

NaN: not a number. CI: confidence interval

## Discussion

The four genes of interest, *CYP21A2*, *CYP17A1*, *HSD3B2* and *CYP11A1*, were expressed at the mRNA level in canine adrenal tissue, allowing PCR amplification, cloning and expression of recombinant radiolabelled proteins for use in serology testing of dogs affected with hypoadrenocorticism. With the exception of a small number of non-synonymous single nucleotide polymorphisms that did not affect the predicted protein sequence, the constructs matched the coding sequences of the respective genes in the CanFam 3.1 dog genome assembly. When expressed, the canine radiolabelled recombinant proteins were of the anticipated size, with the exception of 17-OH, where a second, smaller, band was also seen. The in vitro transcription and translation system can mis-prime on alternative start-codons (ATG, encoding methionine), creating nonsense (if out-of-frame) or truncated (if in-frame) proteins, which might account for this second band. There are 29 possible alternative start codons in the cloned sequence, one of which (starting at position +304) is in frame with the intended start codon and would generate a truncated protein (amino acids 102–508) of approximately 45 kDa, consistent with the observed additional band.

The choice of this particular radioimmunoprecipitation assay for the present study was primarily based on its routine use for human autoantibody serology testing, with its high sensitivity and specificity allowing relatively low levels of autoantibody to be detected [49]. There was also previous experience of using this radioimmunoassay for detecting autoantibodies in sera from diabetic dogs [41]. It has been reported that canine 21-OH autoantibodies can be detected in serum samples by ELISA and that approximately 20–30% of dogs affected with hypoadrenocorticism are autoantibody positive [50]. It was therefore anticipated that a proportion of the hypoadrenocorticoid dogs selected for screening would demonstrate immunoreactivity to 21-OH. However, none of the 20 sera from the dogs affected with hypoadrenocorticism assessed showed any reactivity above that seen with the control dog samples. Although the radioimmunoprecipitation assay used in the present study would be expected to have better reliability for detecting low titre autoantibodies, there are potential technical issues in terms of production of the recombinant protein used in the assay. Misfolding or aggregation of the recombinant protein might interfere with antibody binding to conformational epitopes. Cytochrome P450 enzymes have a tendency to aggregate, due to their hydrophobic membrane associated region [51,52]. However, a comparable human 21-OH construct was used alongside the canine assay to screen sera from autoantibody positive and negative AAD patients (with thanks to Simon Pearce, University of Newcastle), which indicated that the assay was technically sound. Alternatively, it is possible that the difference in 21-OH autoantibody reactivity is related to differences in the case populations in the UK compared to the USA, with different environmental or breed factors affecting autoantibody reactivity to this particular antigen, although this is considered unlikely.

There was a lack of immunoreactivity seen with sera from affected dogs against canine recombinant 17-OH and 3βHSD, autoantibodies against these particular adrenal antigens are less frequently found in human AAD patients compared with 21-OH [11,46,47]. The most interesting finding from the initial autoantibody screen was the immunoreactivity to canine P450scc in a proportion of cases (5/20). The P450scc autoantibody assay was repeated with the same serum samples and this finding was consistent. The presence of such autoantibodies argues in favour of an immune-mediated pathogenesis in canine hypoadrenocorticism and implicates P450scc as a potential target for the autoimmune response against the adrenocortical tissue.

P450scc is the rate-limiting enzyme in the synthesis of all steroid enzymes and is associated with the inner mitochondrial membrane [53]. In mammalian species, it is expressed predominantly in the adrenal gland, ovaries and placenta, with relatively lower expression in the testes [53,54]. In humans, there is also limited expression in mammary tissue, cervix, liver, uterus and lung with small amounts in the skin and vagina [55], as well as in parts of the brain [56]. P450scc autoantibodies are uncommon in healthy humans [47,57–59] with the highest prevalence detected in female patients suffering from ADD and premature ovarian failure (POF) [46,47,59,60], followed by patients with APS [46,57,58,60–64].

When a larger population of dogs affected with hypoadrenocorticism was assessed, 51/213 (24%) were positive for P450scc antibodies. A wider range of diagnostic criteria were accepted for inclusion in the P450scc autoantibody analysis than was used in the initial autoantibody screening part of the study, although this population was largely an extension of the population reported in genetic studies of canine hypoadrenocorticism [17–19]. There was no significant difference in P450scc autoantibody prevalence comparing the 150 dogs that had been selected on the basis of ACTH stimulation test results with the 63 dogs with a historical diagnosis, where samples were submitted for monitoring of steroid replacement therapy. There was no evidence that the time between diagnosis and sampling had a significant effect on the autoantibody prevalence. A population of dogs with more rigorously characterised hypoadrenocorticism, including access to full history, haematology and biochemistry results, aldosterone and ACTH concentrations would be extremely valuable for research. However, the passive nature of sample recruitment for this study precluded such a strategy.

The majority of serum samples from non-hypoadrenocorticoid dogs assessed were classified as P450scc autoantibody negative (*n* = 170) with only two dogs considered to be positive (1.1%). This large population of negative dogs across a range of breeds, ages and diseases, including those with other types of adrenal pathology, increases confidence that the P450scc autoantibody reactivity in dogs affected with hypoadrenocorticism is biologically significant. This prevalence in non-hypoadrenocorticoid dogs is similar to that reported in healthy human populations [47,57–59].

The hospital control case that was weakly positive for P450scc antibody might simply represent a false positive. This dog was affected by a sinonasal epithelial cell tumour, which had infiltrated the frontal cortex. Such tumours can be locally invasive as well as metastatic [65], including metastasis to the adrenal glands [66]. It is therefore conceivable that the source of P450scc antigenic stimulation in this dog might be from adrenal metastatic disease or from within the brain itself, as P450scc is expressed in the cortex, the basal ganglia and, to a lesser extent, in the olfactory bulb [56]. The patient classified as P450scc autoantibody positive within the group of dogs affected with hyperadrenocorticism demonstrated P450scc immunoreactivity well above the threshold. The sample from this dog was submitted for monitoring of trilostane therapy, but with no further information or history provided. Since it is estimated that 15–20% of dogs affected with hyperadrenocorticism have adrenal dependent disease [67], it is possible that this miniature poodle developed P450scc antibodies as a result of antigenic stimulation associated with its adrenal pathology.

The age of onset of hypoadrenocorticism in this study population was consistent with that reported in the literature with a range of 3 months to 11 years with a median of 4 years and 5 months [5,8]. There was no pronounced sex bias in the study population (53.5% female) although a female predisposition to canine hypoadrenocorticism has been reported [5]. This might reflect the heterogeneous breed profile of dogs in the present study, compared with other studies that have focussed on individual breeds. There was a significantly higher proportion of female dogs positive for P450scc autoantibodies (30%) compared with males (17%), regardless of neuter status. Whilst there is no apparent sex predisposition for hypothyroidism or thyroglobulin autoantibody status in dogs [32], females are more likely to have thyroid hormone autoantibodies than male dogs [38]. In humans, there is a gender bias towards women being positive for P450scc autoantibodies, and an association with premature ovarian failure (POF). It is possible that male dogs are less likely to make P450scc autoantibodies than female dogs, or that male and female dogs are equally reactive but that females have enhanced immune stimulation from additional or persistent P450scc expressed by the female reproductive tract, which maintains production of the autoantibodies, whereas in males, the autoantibodies wane more quickly as the disease progresses from lymphocytic adrenalitis to adrenocortical atrophy. Further investigation of reproductive parameters in female entire dogs affected with hypoadrenocorticism is warranted, with anecdotal evidence suggesting failure of normal cycling may be present in a proportion of the breeding bitches. As a large proportion of dogs in the UK are neutered and female entire dogs affected with hypoadrenocorticism are not usually used for breeding purposes, this could be an under-recognised problem [68].

Despite some dog breeds reported to be more susceptible to hypoadrenocorticism, the wide range of breeds represented in the present study is consistent with other studies [5,6,8]. Crossbreed dogs were the most commonly represented in the study population, with West Highland white terriers, poodles and springer spaniels also highly represented. Some breeds with known genetic heritability [4,69] were not particularly represented, including the Nova Scotia duck tolling retriever (*n* = 0) and Portuguese water dog (*n* = 2), probably reflective of breed preferences in the UK. Of the other two breeds with a known genetic heritability, only one of 11 standard poodles was P450scc antibody positive and none of the five bearded collies assessed were autoantibody positive. Despite the relatively large case population, the numbers were low once stratified for breed, however, breed was significantly associated with P450scc autoantibody status. Although there were no autoantibody positive Border terriers (*n* = 5), Weimaraners (*n* = 6) or Rottweilers (*n* = 4), it is important to note that the numbers assessed were relatively small. Three of seven Border collies were P450scc autoantibody positive and a further four of eight dogs reported simply as ‘collie’ were positive, suggesting that there might be an increased prevalence of P450scc autoantibodies in collie-type dogs affected with hypoadrenocorticism.

Breed differences in susceptibility to hypoadrenocorticism and P450scc autoantibody prevalence suggest there might be underlying genetic factors. Given the importance of MHC genes in autoantibody reactivity in human AAD [2,70] and an association between DLA-type and canine hypoadrenocorticism [17,21], the relationship between DLA alleles and P450scc autoantibody status was explored. A large number of DLA haplotypes were represented in the case population, reflecting the breed diversity present [71]. DLA-DQA1*006:01 was identified as a potential risk allele for P450scc autoantibody reactivity. DLA-DQA1*006:01 has also been found to be associated with disease risk for hypoadrenocorticism in springer spaniels, standard poodles [17] and Nova Scotia duck tolling retrievers (NSDTRs) [21], as part of the DLA-DRB1*015—DQA1*006—DQB1*023 haplotype. It remains unclear whether the DQA1 association is due to a more restricted set of alleles present in this study compared with DRB1 and DQB1, allowing significant associations to be identified, or whether there is a DQA1-specific effect on disease risk and/or autoantibody status. There are only two previous reports linking autoantibody status and DLA-type. In hypothyroidism, DQA1*001:01 is more frequently found in dogs with thyroglobulin autoantibodies (OR = 2.57; 95% confidence interval = 1.28–5.17) [72]. Also, DLA-DRB1*006:01—DQA1*005:01:1—DQB1*020:01 is associated with increased antinuclear antibody reactivity in NSDTRs [73]. The current study therefore represents one of the few studies investigating the genetic basis of the autoimmune response in dogs with spontaneous disease.

## Conclusion

This study is the first description of specific autoantibodies in canine hypoadrenocorticism, demonstrating the presence of P450scc autoantibodies in a proportion of dogs affected with hypoadrenocorticism. P450scc autoantibodies are more prevalent in affected female dogs and the P450scc autoantibody status appears to be related to breed and DLA-type. Further work is required to determine whether the presence of P450scc autoantibodies is associated with reproductive dysfunction in affected female dogs and whether measurement of circulating P450scc autoantibodies is of use as part of the diagnostic approach for canine hypoadrenocorticism.

## Supporting Information

S1 Appendix Primers for amplification of canine adrenal autoantigen genes. (DOCX)

S1 Fig Original gel image for Fig 1A. (TIF)

## References

[1] Summers JF, Diesel G, Asher L, McGreevy PD, Collins LM (2010) Inherited defects in pedigree dogs. Part 2: Disorders that are not related to breed standards. The Veterinary Journal 183: 39–45. 10.1016/j.tvjl.2009.11.002 19963415

[2] Mitchell AL, Pearce SHS (2012) Autoimmune Addison disease: pathophysiology and genetic complexity. Nature reviews Endocrinology 8: 306–316. 10.1038/nrendo.2011.245 22290360

[3] Betterle C, Dal Pra C, Mantero F, Zanchetta R (2002) Autoimmune adrenal insufficiency and autoimmune polyendocrine syndromes: autoantibodies, autoantigens, and their applicability in diagnosis and disease prediction. Endocrine Reviews 23: 327–364. 12050123 10.1210/edrv.23.3.0466

[4] Hughes AM, Nelson RW, Famula TR, Bannasch DL (2007) Clinical features and heritability of hypoadrenocorticism in Nova Scotia Duck Tolling Retrievers: 25 cases (1994–2006). Journal of the American Veterinary Medical Association 231: 407–412. 17669043 10.2460/javma.231.3.407

[5] Scott-Moncrieff C (2015) Hypoadrenocorticism In: Feldman EC, Nelson RW, editors. Canine and Feline Endocrinology. 4 ed: Elselvier pp. 485–520.

[6] Peterson ME, Kintzer PP, Kass PH (1996) Pretreatment clinical and laboratory findings in dogs with hypoadrenocorticism: 225 cases (1979–1993). Journal of the American Veterinary Medical Association 208: 85–91. 8682712

[7] Thompson AL, Scott-Moncrieff JCR, Anderson JD (2007) Comparison of classic hypoadrenocorticism with glucocorticoid-deficient hypoadrenocorticism in dogs: 46 cases (1985–2005). Journal of the American Veterinary Medical Association 230: 1190–1194. 17501661 10.2460/javma.230.8.1190

[8] Kelch WJ (1996) Canine Hypoadrenocorticism (Canine Addison's Disease): History, Contemporary Diagnosis by Practicing Veterinarians, and Epidemiology. Knoxville: University of Tennessee.

[9] Adler JA, Drobatz KJ, Hess RS (2007) Abnormalities of serum electrolyte concentrations in dogs with hypoadrenocorticism. Journal of Veterinary Internal Medicine / American College of Veterinary Internal Medicine 21: 1168–1173. 10.1892/06-270.1 18196721

[10] Watson AD, Church DB, Emslie DR, Foster SF (1998) Plasma cortisol responses to three corticotrophic preparations in normal dogs. Australian Veterinary Journal 76: 255–257. 9612546 10.1111/j.1751-0813.1998.tb10153.x

[11] Betterle C, Coco G, Zanchetta R (2005) Adrenal cortex autoantibodies in subjects with normal adrenal function. Best Practice & Research Clinical Endocrinology & Metabolism 19: 85–99. 15826924 10.1016/j.beem.2004.11.008

[12] Erichsen MM, Løvås K, Skinningsrud B, Wolff AB, Undlien DE, et al (2009) Clinical, immunological, and genetic features of autoimmune primary adrenal insufficiency: observations from a Norwegian registry. The Journal of Clinical Endocrinology and Metabolism 94: 4882–4890. 10.1210/jc.2009-1368 19858318

[13] Bellumori TP, Famula TR, Bannasch DL, Belanger JM, Oberbauer AM (2013) Prevalence of inherited disorders among mixed-breed and purebred dogs: 27,254 cases (1995–2010). Journal of the American Veterinary Medical Association 242: 1549–1555. 10.2460/javma.242.11.1549 23683021

[14] Boag AM, Catchpole B (2014) A Review of the Genetics of Hypoadrenocorticism. Topics in Companion Animal Medicine 29: 96–101. 10.1053/j.tcam.2015.01.001 25813849

[15] Chase K, Sargan D, Miller K, Ostrander EA, Lark KG (2006) Understanding the genetics of autoimmune disease: two loci that regulate late onset Addison's disease in Portuguese Water Dogs. International Journal of Immunogenetics 33: 179–184. 16712648 10.1111/j.1744-313X.2006.00593.x PMC2775482

[16] Famula TR, Belanger JM, Oberbauer AM (2003) Heritability and complex segregation analysis of hypoadrenocorticism in the standard poodle. The Journal of Small Animal Practice 44: 8–12. 12570345 10.1111/j.1748-5827.2003.tb00096.x

[17] Massey J, Boag A, Short AD, Scholey RA, Henthorn PS, et al (2013) MHC class II association study in eight breeds of dog with hypoadrenocorticism. Immunogenetics 65: 291–297. 10.1007/s00251-013-0680-2 23358933

[18] Short AD, Catchpole B, Boag AM, Kennedy LJ, Massey J, et al (2014) Putative candidate genes for canine hypoadrenocorticism (Addison's disease) in multiple dog breeds. Vet Rec 175: 430 10.1136/vr.102160 25124887

[19] Short AD, Boag A, Catchpole B, Kennedy LJ (2013) A Candidate Gene Analysis of Canine Hypoadrenocorticism in 3 Dog Breeds. The Journal of Heredity 104: 807–820. 10.1093/jhered/est051 23997205

[20] Hughes AM, Bannasch DL, Kellett K, Oberbauer AM (2011) Examination of candidate genes for hypoadrenocorticism in Nova Scotia Duck Tolling Retrievers. The Veterinary Journal 187: 212–216. 10.1016/j.tvjl.2009.10.012 19931476

[21] Hughes AM, Jokinen P, Bannasch DL, Lohi H, Oberbauer AM (2010) Association of a dog leukocyte antigen class II haplotype with hypoadrenocorticism in Nova Scotia Duck Tolling Retrievers. Tissue Antigens 75: 684–690. 10.1111/j.1399-0039.2010.01440.x 20136772

[22] Hadlow WJ (1953) Adrenal cortical atrophy in the dog; report of three cases. The American Journal of Pathology 29: 353–361. 13030696 PMC1937395

[23] Schaer M, Riley WJ, Buergelt CD, Bowen DJ, Senior DF, et al (1986) Autoimmunity and Addison's disease in the dog. Journal of the American Animal Hospital Association 22: 789–794.

[24] Boujon CE, Bornand-Jaunin V, Schärer V, Rossi GL, Bestetti GE (1994) Pituitary gland changes in canine hypoadrenocorticism: a functional and immunocytochemical study. Journal of Comparative Pathology 111: 287–295. 7836570 10.1016/s0021-9975(05)80007-6

[25] Adissu HA, Hamel-Jolette A, Foster RA (2010) Lymphocytic Adenohypophysitis and Adrenalitis in a Dog With Adrenal and Thyroid Atrophy. Veterinary Pathology 47: 1082–1085. 10.1177/0300985810382520 20807820

[26] Frank CB, Valentin SY, Scott-Moncrieff JCR, Miller MA (2013) Correlation of Inflammation with Adrenocortical Atrophy in Canine Adrenalitis. Journal of Comparative Pathology 149: 268–279. 10.1016/j.jcpa.2012.11.242 23348017

[27] Bowen D, Schaer M, Riley W (1986) Autoimmune polyglandular syndrome in a dog: a case report. The Journal of the American Animal Hospital Association 22: 649–654.

[28] Blizzard RM, Kyle M (1963) Studies of the adrenal antigens and antibodies in Addison's disease. The Journal of Clinical Investigation 42: 1653–1660. 14074360 10.1172/JCI104851 PMC289445

[29] Rose NR, Bona C (1993) Defining criteria for autoimmune diseases (Witebsky's postulates revisited). Immunology Today 14: 426–430. 8216719 10.1016/0167-5699(93)90244-F

[30] Lleo A, Invernizzi P, Bin Gao B, Podda M, Gershwin ME (2010) Definition of human autoimmunity—autoantibodies versus autoimmune disease. Autoimmunity Reviews 9: A259–A266. 10.1016/j.autrev.2009.12.002 19963079

[31] Patzl M, Möstl E (2003) Determination of autoantibodies to thyroglobulin, thyroxine and triiodothyronine in canine serum. Journal of Veterinary Medicine A, Physiology, pathology, clinical medicine 50: 72–78. 12667197 10.1046/j.1439-0442.2003.00491.x

[32] Dixon RM, Mooney CT (1999) Canine serum thyroglobulin autoantibodies in health, hypothyroidism and non-thyroidal illness. Research in Veterinary Science 66: 243–246. 10333466 10.1053/rvsc.1998.0268

[33] Nachreiner RF, Refsal KR, Graham PA, Hauptman J, Watson GL (1998) Prevalence of autoantibodies to thyroglobulin in dogs with nonthyroidal illness. American Journal of Veterinary Research 59: 951–955. 9706197

[34] Piechotta M, Arndt M, Hoppen HO (2010) Autoantibodies against thyroid hormones and their influence on thyroxine determination with chemiluminescence immunoassay in dogs. Journal of Veterinary Science 11: 191–196. 20706025 10.4142/jvs.2010.11.3.191 PMC2924479

[35] Skopek E, Patzl M, Nachreiner RF (2006) Detection of autoantibodies against thyroid peroxidase in serum samples of hypothyroid dogs. American Journal of Veterinary Research 67: 809–814. 16649914 10.2460/ajvr.67.5.809

[36] McLachlan SM, Rapoport B (2007) Thyroid peroxidase as an autoantigen. Thyroid: Official Journal of the American Thyroid Association 17: 939–948. 17822378 10.1089/thy.2007.0169

[37] Graham PA, Refsal KR, Nachreiner RF (2007) Etiopathologic Findings of Canine Hypothyroidism. Veterinary Clinics of North America: Small Animal Practice 37: 617–631. 17619002 10.1016/j.cvsm.2007.05.002

[38] Nachreiner RF, Refsal KR, Graham PA, Bowman MM (2002) Prevalence of serum thyroid hormone autoantibodies in dogs with clinical signs of hypothyroidism. Journal of the American Veterinary Medical Association 220: 466–471. 11860240 10.2460/javma.2002.220.466

[39] Davison LJ (2004) Canine diabetes mellitus: a clinical and immunological study London: University of London.

[40] Davison LJ, Herrtage ME, Catchpole B (2011) Autoantibodies to recombinant canine proinsulin in canine diabetic patients. Research in Veterinary Science 91: 58–63. 10.1016/j.rvsc.2010.08.007 20855094

[41] Davison LJ, Weenink SM, Christie MR, Herrtage ME, Catchpole B (2008) Autoantibodies to GAD65 and IA-2 in canine diabetes mellitus. Veterinary Immunology and Immunopathology 126: 83–90. 10.1016/j.vetimm.2008.06.016 18706702

[42] Devendra D, Yu L, Eisenbarth GS (2004) Endocrine autoantibodies. Clinics in Laboratory Medicine 24: 275–303. 15157566 10.1016/j.cll.2004.01.012

[43] Anderson JR, Goudie RB, Gray KG, Timbury GC (1957) Auto-antibodies in Addison's disease. Lancet 272: 1123–1124. 13439984 10.1016/s0140-6736(57)91687-2

[44] Bednarek J, Furmaniak J, Wedlock N, Kiso Y, Baumann-Antczak A, et al (1992) Steroid 21-hydroxylase is a major autoantigen involved in adult onset autoimmune Addison's disease. FEBS letters 309: 51–55. 1511745 10.1016/0014-5793(92)80737-2

[45] Husebye E, Løvås K (2009) Pathogenesis of primary adrenal insufficiency. Best Practice and Research Clinical Endocrinology and Metabolism 23: 147–157. 10.1016/j.beem.2008.09.004 19500759

[46] Betterle C, Volpato M, Pedini B, Chen S, Smith BR, et al (1999) Adrenal-cortex autoantibodies and steroid-producing cells autoantibodies in patients with Addison's disease: comparison of immunofluorescence and immunoprecipitation assays. The Journal of Clinical Endocrinology and Metabolism 84: 618–622. 10022426 10.1210/jcem.84.2.5459

[47] Falorni A, Laureti S, Candeloro P, Perrino S, Coronella C, et al (2002) Steroid-cell autoantibodies are preferentially expressed in women with premature ovarian failure who have adrenal autoimmunity. Fertility and Sterility 78: 270–279. 12137862 10.1016/s0015-0282(02)03205-3

[48] Razali NM, Wah YB (2011) Power comparisons of shapiro-wilk, kolmogorov-smirnov, lilliefors and anderson-darling tests. Journal of Statistical Modeling and Analytics 2: 21–33.

[49] Falorni A, Chen S, Zanchetta R, Yu L, Tiberti C, et al (2011) Measuring adrenal autoantibody response: Interlaboratory concordance in the first international serum exchange for the determination of 21-hydroxylase autoantibodies. Clinical Immunology 140: 291–299. 10.1016/j.clim.2011.04.012 21570358

[50] Rick M, Refsal KR, Callewaert DM, Rader T. The measurement of 21-hydroxylase antibodies in dogs via enzyme-linked immunosorbent assay; 2013; Liverpool, UK.

[51] Scott EE, Spatzenegger M, Halpert JR (2001) A Truncation of 2B Subfamily Cytochromes P450 Yields Increased Expression Levels, Increased Solubility, and Decreased Aggregation While Retaining Function. Archives of Biochemistry and Biophysics 395: 57–68. 11673866 10.1006/abbi.2001.2574

[52] Arase M, Waterman MR, Kagawa N (2006) Purification and characterization of bovine steroid 21-hydroxylase (P450c21) efficiently expressed in Escherichia coli. Biochemical and Biophysical Research Communications 344: 400–405. 16597434 10.1016/j.bbrc.2006.03.067

[53] Miller WL, Auchus RJ (2011) The Molecular Biology, Biochemistry, and Physiology of Human Steroidogenesis and Its Disorders. Endocrine Reviews 32: 81–151. 10.1210/er.2010-0013 21051590 PMC3365799

[54] Luu-The V, Pelletier G, Labrie F (2005) Quantitative appreciation of steroidogenic gene expression in mouse tissues: new roles for type 2 5alpha-reductase, 20alpha-hydroxysteroid dehydrogenase and estrogen sulfotransferase. The Journal of Steroid Biochemistry and Molecular Biology 93: 269–276. 15860270 10.1016/j.jsbmb.2005.01.003

[55] Luu-The V (2013) Assessment of steroidogenesis and steroidogenic enzyme functions. The Journal of Steroid Biochemistry and Molecular Biology: 176–182. 10.1016/j.jsbmb.2013.05.017 23770321

[56] Mellon SH, Griffin LD, Compagnone NA (2001) Biosynthesis and action of neurosteroids. Brain Research Brain research reviews 37: 3–12. 11744070 10.1016/s0165-0173(01)00109-6

[57] Uibo R, Perheentupa J, Ovod V, Krohn KJ (1994) Characterization of adrenal autoantigens recognized by sera from patients with autoimmune polyglandular syndrome (APS) type I. Journal of Autoimmunity 7: 399–411. 7916911 10.1006/jaut.1994.1029

[58] Reimand K, Peterson P, Hyöty H, Uibo R, Cooke I, et al (2000) 3beta-hydroxysteroid dehydrogenase autoantibodies are rare in premature ovarian failure. The Journal of Clinical Endocrinology and Metabolism 85: 2324–2326. 10852471 10.1210/jcem.85.6.6630

[59] De Carmo Silva R, Kater CE, Dib SA, Laureti S, Forini F, et al (2000) Autoantibodies against recombinant human steroidogenic enzymes 21-hydroxylase, side-chain cleavage and 17alpha-hydroxylase in Addison's disease and autoimmune polyendocrine syndrome type III. European Journal of Endocrinology / European Federation of Endocrine Societies 142: 187–194. 10664529 10.1530/eje.0.1420187

[60] Pra CD, Chen S, Furmaniak J, Smith BR, Pedini B, et al (2003) Autoantibodies to steroidogenic enzymes in patients with premature ovarian failure with and without Addison's disease. European Journal of Endocrinology / European Federation of Endocrine Societies 148: 565–570. 12720541 10.1530/eje.0.1480565

[61] Cihakova D, Trebusak K, Heino M, Fadeyev V, Tiulpakov A, et al (2001) Novel AIRE mutations and P450 cytochrome autoantibodies in Central and Eastern European patients with APECED. Human Mutation 18: 225–232. 11524733 10.1002/humu.1178

[62] Peterson P, Uibo R, Peränen J, Krohn K (1997) Immunoprecipitation of steroidogenic enzyme autoantigens with autoimmune polyglandular syndrome type I (APS I) sera; further evidence for independent humoral immunity to P450c17 and P450c21. Clinical and Experimental Immunology 107: 335–340. 9030872 10.1111/j.1365-2249.1997.282-ce1175.x PMC1904569

[63] Myhre AG, Halonen M, Eskelin P, Ekwall O, Hedstrand H, et al (2001) Autoimmune polyendocrine syndrome type 1 (APS I) in Norway. Clinical Endocrinology 54: 211–217. 11207636 10.1046/j.1365-2265.2001.01201.x

[64] Perniola R, Falorni A, Clemente MG, Forini F, Accogli E, et al (2000) Organ-specific and non-organ-specific autoantibodies in children and young adults with autoimmune polyendocrinopathy-candidiasis-ectodermal dystrophy (APECED). European Journal of Endocrinology / European Federation of Endocrine Societies 143: 497–503. 11022196 10.1530/eje.0.1430497

[65] Patnaik AK (1989) Canine sinonasal neoplasms: clinicopathological study of 285 cases. Journal of the American Animal Hospital Association v. 25(1) p. 103–113.

[66] Labelle P, De Cock HEV (2005) Metastatic tumors to the adrenal glands in domestic animals. Veterinary Pathology 42: 52–58. 15657272 10.1354/vp.42-1-52

[67] Kooistra HS, Galac S (2012) Recent Advances in the Diagnosis of Cushing's Syndrome in Dogs. Topics in Companion Animal Medicine 27: 21–24. 10.1053/j.tcam.2012.06.001 22958793

[68] O’Neill DG, Church DB, McGreevy PD, Thomson PC, Brodbelt DC (2013) Longevity and mortality of owned dogs in England. The Veterinary Journal 198: 638–643. 10.1016/j.tvjl.2013.09.020 24206631

[69] Oberbauer AM, Bell JS, Belanger JM, Famula TR (2006) Genetic evaluation of Addison's disease in the Portuguese Water Dog. BMC Veterinary Research 2: 15 16670022 10.1186/1746-6148-2-15 PMC1481556

[70] Baker PR, Baschal EE, Fain PR, Nanduri P, Triolo TM, et al (2011) Dominant suppression of Addison's disease associated with HLA-B15. The Journal of Clinical Endocrinology and Metabolism 96: 2154–2162. 10.1210/jc.2010-2964 21565792 PMC3135206

[71] Kennedy LJ, Barnes A, Happ GM, Quinnell RJ, Bennett D, et al (2002) Extensive interbreed, but minimal intrabreed, variation of DLA class II alleles and haplotypes in dogs. Tissue Antigens 59: 194–204. 12074709 10.1034/j.1399-0039.2002.590303.x

[72] Kennedy LJ, Quarmby S, Happ GM, Barnes A, Ramsey IK, et al (2006) Association of canine hypothyroidism with a common major histocompatibility complex DLA class II allele. Tissue Antigens 68: 82–86. 16774545 10.1111/j.1399-0039.2006.00614.x

[73] Wilbe M, Andersson G (2012) MHC class II is an important genetic risk factor for canine systemic lupus erythematosus (SLE)-related disease: implications for reproductive success. Zuchthygiene (Reproduction in Domestic Animals) 47 Suppl 1: 27–30. 22212209 10.1111/j.1439-0531.2011.01962.x

