# Supplementary material for: Autoantibodies against Cytochrome P450 Side-Chain Cleavage Enzyme in Dogs (Canis lupus familiaris) Affected with Hypoadrenocorticism (Addison’s Disease)
Source: PLoS One. 2015 Nov 30;10(11):e0143458. doi: 10.1371/journal.pone.0143458 (PMC4664467; doi:10.1371/journal.pone.0143458)
Supplement: S1 Appendix — (DOCX) [file pone.0143458.s001.docx]

**S1 Appendix**

Table 1. Primers for amplification of canine adrenal autoantigen genes.

| Primer | Sense sequence  (5ʹ-3ʹ) | Antisense sequence  (5ʹ-3ʹ) | Amplicon size (bp) |
| --- | --- | --- | --- |
| CYP21A2scr | CGCCTGGGGCTGCAAGATGT | AGATGATGGCGCAGGTGAGCA | 334 |
| CYP21A2clo | *AAGCTT*GCCACC**ATG**CTACTCCTCGGAGTG | *CTCGAG***TCA**TGGGTGCTGGCCACGTC | 1497 |
| CYP17A1scr | CAGCAGCGACTCTATCACCA | ATGCGAAGCACCTCTCTGAT | 336 |
| CYP17A1clo | *CTCGAG*CCACC**ATG**TGGGAACTCTTG | *CTCGAG***TCA**TGGGTGCTGGCCACGTC | 1872 |
| CYP11A1scr | TCCCGCCAAGACGTTGGTGC | GCGGGGAGGGAAAAGGTGGC | 557 |
| CYP11A1clo* | *AAGCTT*AGGGACGGTAGCGATCGTGG | *TCTAGA*GGAGGGAAAAGGTGGCGCGA | 1829 |
| HSD3B2scr | GGAGATCCGGGCACTGGACAAA | CCAGCGCCAATCCACTCCGTGA | 992 |
| HSD3B2clo | *GGTACC*GCCACC**ATG**GCTGGATGGAGCTGC | *TCTAGA*CCATCCTCAGA**TCA**GTGAGTC |  |

Bases in italics are restriction sites introduced for directional subcloning (HindIII: AAGCTT; XhoI: CTCGAG; XbaI: TCTAGA; KpnI: GGTACC). Start and stop codons are shown bold and underlined. * primers located in 5’ UTR (sense) and 3’ UTR (antisense).
